# Reliability and day-to-day variability of peak fat oxidation during treadmill ergometry

**DOI:** 10.1186/s12970-016-0115-1

**Published:** 2016-01-25

**Authors:** Raul De Souza Silveira, Anja Carlsohn, Georg Langen, Frank Mayer, Friederike Scharhag-Rosenberger

**Affiliations:** University Outpatient Clinic, Center of Sports Medicine, Potsdam University, Potsdam, Germany; Swiss Federal Institute of Sport Magglingen, Magglingen, Switzerland; Department of Health Sciences, University of Education Schwaebisch Gmuend, Schwaebisch Gmuend, Germany; Department of Medical Oncology, National Center for Tumor Diseases (NCT), Heidelberg University Hospital, Heidelberg, Germany

**Keywords:** Peak fat oxidation, Reliability, Variability, Running, Treadmill ergometry

## Abstract

**Background:**

Exercising at intensities where fat oxidation rates are high has been shown to induce metabolic benefits in recreational and health-oriented sportsmen. The exercise intensity (Fat_peak_) eliciting peak fat oxidation rates is therefore of particular interest when aiming to prescribe exercise for the purpose of fat oxidation and related metabolic effects. Although running and walking are feasible and popular among the target population, no reliable protocols are available to assess Fat_peak_ as well as its actual velocity (V_PFO_) during treadmill ergometry. Our purpose was therefore, to assess the reliability and day-to-day variability of V_PFO_ and Fat_peak_ during treadmill ergometry running.

**Methods:**

Sixteen recreational athletes (f = 7, m = 9; 25 ± 3 y; 1.76 ± 0.09 m; 68.3 ± 13.7 kg; 23.1 ± 2.9 kg/m^2^) performed 2 different running protocols on 3 different days with standardized nutrition the day before testing. At day 1, peak oxygen uptake (VO_2peak_) and the velocities at the aerobic threshold (V_LT_) and respiratory exchange ratio (RER) of 1.00 (V_RER_) were assessed. At days 2 and 3, subjects ran an identical submaximal incremental test (Fat-peak test) composed of a 10 min warm-up (70 % V_LT_) followed by 5 stages of 6 min with equal increments (stage 1 = V_LT_, stage 5 = V_RER_). Breath-by-breath gas exchange data was measured continuously and used to determine fat oxidation rates. A third order polynomial function was used to identify V_PFO_ and subsequently Fat_peak_. The reproducibility and variability of variables was verified with an intraclass correlation coefficient (ICC), Pearson’s correlation coefficient, coefficient of variation (CV) and the mean differences (bias) ± 95 % limits of agreement (LoA).

**Results:**

ICC, Pearson’s correlation and CV for V_PFO_ and Fat_peak_ were 0.98, 0.97, 5.0 %; and 0.90, 0.81, 7.0 %, respectively. Bias ± 95 % LoA was −0.3 ± 0.9 km/h for V_PFO_ and −2 ± 8 % of VO_2peak_ for Fat_peak_.

**Conclusion:**

In summary, relative and absolute reliability indicators for V_PFO_ and Fat_peak_ were found to be excellent. The observed LoA may now serve as a basis for future training prescriptions, although fat oxidation rates at prolonged exercise bouts at this intensity still need to be investigated.

## Background

Fat is next to carbohydrate the main substrate to fuel prolonged endurance exercise over a wide range of intensities. Exercising at intensities where fat oxidation rates are high has been advocated to induce metabolic changes that benefit both professional and recreational endurance athletes, as well as health-oriented exercisers [1]. The oxidative regulation of fat metabolism is intricate and may be influenced by the intensity, duration and type of the activity, as well as dietary intake pattern, muscle glycogen concentrations, gender and training status [2–6]. When described as a sole function of exercise intensity, fat oxidation will augment as intensity increases from low to moderate levels, achieving peak oxidation rates between 45 and 65 % of peak oxygen uptake (VO_2peak_), then to become minimal at intensities above 85 % of VO_2peak_ [1, 7–9].

In recent years, there has been an emerging interest involving the maximization of fat metabolism during exercise (e.g. with the aim of improving athletic training, generally related to performance enhancement aspects in athletes or linked to therapeutic effects in patients) [10, 11]. Consequently, reliably identifying the intensity at which fat metabolism reaches peak oxidation levels is crucial when prescribing exercise for the purpose of fat oxidation and related metabolic effects [12]. The reproducibility of the intensity eliciting peak fat oxidation (PFO) rates (i.e. Fat_peak_, but also referred to as Fat_max_ or LIPOXmax) has been reported for a variety of submaximal incremental protocols [6, 7, 13–16]. However, all reliability studies to date have used cycle ergometry as the exercising method of choice, which in turn may limit a valid transferability from any of the previously tested protocols and their respective reproducibility indicators into other types of exercise. Yet, despite running and walking being feasible and popular modalities among different target populations [17], there are to date no reliability data on the estimations of Fat_peak_ during treadmill ergometry. Additionally, only a few studies have performed comprehensive statistical assessments as recommended by the guidelines for reliability assessment in sports medicine [18]. These would include for instance, the establishment of both relative and absolute reliability indicators for key variables related to Fat_peak_ estimations, such as the actual velocity at which PFO rates occur (i.e. V_PFO_), as well as the computation of its respective intrasubject (day-to-day) variability. Therefore, the aims of the current investigation were to establish the reproducibility of V_PFO_ and Fat_peak_, and therewith contribute to the improvement of training prescriptions in running to enhance fat metabolism.

## Methods

### Subjects

Sixteen healthy and active adults involved in the regular practice of different sports disciplines (i.e. running, cycling, rugby and weight-lifting) voluntarily took part in the present investigation. The study was conducted in accordance with the declaration of Helsinki. The ethics committee from Potsdam University approved the study and participants gave their written informed consent after receiving detailed information about the investigational protocol and aims. Inclusion criterion was ≥3 h of training per week. The participants’ anthropometric and training data are given in Table 1.

**Table 1:** Anthropometric and training data of subjects

	Overall (*n* = 16)	Males (*n* = 9)	Females (*n* = 7)
Age (yrs.)	25 ± 3	26 ± 3	23 ± 2^*^
Height (m)	1.76 ± 0.09	1.81 ± 0.07	1.69 ± 0.06^*^
Weight (kg)	68.3 ± 13.7	81.9 ± 6.5	59.8 ± 7.1^*^
BMI (kg/m^2^)	23.1 ± 2.9	24.8 ± 1.9	21.0 ± 2.0^*^
%BF	14.2 ± 3.7	12.3 ± 2.3	16.7 ± 2.8^*^
Training (h/week)	7 ± 2	7 ± 3	6 ± 2

All values are mean ± SD; BMI, Body mass index; %BF, Percentage body fat; *P < 0.05 (gender comparisons only)

### General design

All examinations were conducted at Potsdam University’s Outpatient Clinic. At day 1, a full medical check (anamnesis, anthropometrical assessment, physical examination, resting ECG) was carried out preceding the first exercise appointment as recommended by the German Federation for Cardiovascular Prevention and Rehabilitation [19]. Subsequently, participants performed a maximal baseline running test to determine the exercise stages for the Fat-peak tests. On days 2 and 3, an identical submaximal incremental running test (Fat-peak test 1 and 2) was carried out on the same treadmill ergometer (0.4 % inclination) (H/P/ Cosmos Pulsar Graphics. 2005®, Germany). A breath-by-breath Metamax 3B system (Cortex Biophysik GmbH. Leipzig, Germany) was used to monitor respiratory data and to determine lipid oxidation rates via indirect calorimetry. Diet was controlled on the day prior to each of the submaximal tests. Participants performed all tests in a fasted state and were additionally advised to refrain from training during the 24 h before each bout. Female’s menstrual cycle was uncontrolled.

### Baseline test

The baseline test consisted of a stepwise incremental running bout until volitional exhaustion. The initial stage of 6 km/h, stage increments of 2 km/h and stage duration of 3 min were defined to exhaust subjects in not less than 4 stages [15]. Lactate concentrations were measured in between stages from capillary blood samples taken from the hyperemized earlobe (Biosen S line, EKF diagnostic GmbH. Magdeburg, Germany). Subsequently, the following parameters were determined: The velocities at the aerobic threshold (V_LT_) [20] and respiratory exchange ratio (RER) of 1.00 (V_RER_), as well as VO_2peak_ and peak running velocity (V_peak_).

### Fat-peak tests

Forty-eight hours after baseline, subjects performed the first submaximal incremental run. The bout lasted 30 min, i.e. 5 stages of 6 min, and was designed on an individualized basis, based on the recorded gas-exchange and blood lactate variables from each participant [15]. The starting velocity was set at V_LT_ while the end velocity was V_RER_. Hence, to obtain five stages of equal increment, the difference between end- and start-velocity needs to be divided by four (i.e. [(V_RER_ - V_LT_) ÷ 4 = increment]). Before officially commencing the test, a 10 min warm up phase at 70 % V_LT_ was implemented to stabilize cardiopulmonary parameters and reduce possible breathing artifacts that may arise at the beginning of exercise calorimetry [21]. The second (identical) submaximal bout was then carried out 48 to 72 h later at the same time for each participant (07:00, 8:00 or 9:00 am). Subsequently, the following parameters were determined: fat oxidation rates, PFO, V_PFO_, oxygen uptake (VO_2_) at V_PFO_ and heart rate (HR) at V_PFO_.

### Dietary control

For compliance control, food intake was documented in a standardized diet record form [22] during the day before each submaximal run and analyzed later on. Participants were not given any specific dietary recommendations, but simply told to identically repeat their conventional nutritional plan at both days. A 12-h overnight fast was also enforced before every running bout. Nutrient and energetic values, including possible deviations within diet record forms were computed based on the German Nutrition database (PRODI 5.7, Nutri-Science GmbH. Hausach, Germany).

### Gas exchange data analysis

Gas exchange data were checked for plausibility and analyzed using the software Metasoft 3, version 3.9. VO_2peak_ was defined as the highest 30 s average value during the baseline test. For the Fat-peak tests, fat oxidation rates were calculated from VO_2_ and the non-protein RER according to Péronnet [23]. Gas exchange data (viewed with time interval of 10 s) were averaged over the last 30 s of each stage. By applying a third polynomial (P3) function (Prism 6, GraphPad Software Inc.), a graphic depiction of fat oxidation rates as a function of exercise intensity was created for each individual and used to determine PFO, V_PFO_, Fat_peak_ [16, 24] and subsequently VO_2_ and HR at V_PFO_.

### Statistics

All of the analyzed parameters are descriptively reported as mean ± standard deviation (SD). Statistical analysis was performed using SPSS, version 20, IBM, USA & Microsoft Excel 2011. Samples were checked for normality using the Shapiro-Wilk test. Gender differences in anthropometry, training and baseline performance data were tested with an un-paired *t*-test. During the Fat-peak tests, differences in VO_2_, RER, fat oxidation rates and HR were assessed with a two-way ANOVA for repeated measures (test X stage). A paired *t*-test assessed the in between test differences for V_PFO_, PFO, Fat_peak,_ VO_2_ at V_PFO_, HR at V_PFO_, as well as the differences in the dietary data. Relative and absolute reliability of V_PFO_ and Fat_peak_ were verified with an intraclass correlation coefficient (ICC), the coefficient of variation (CV) and the Pearson’s correlation coefficient. The day-to-day variability of V_PFO_ and Fat_peak_ was assessed with a Bland-Altman analysis by establishing the mean differences (bias) ± 95 % limits of agreement (LoA). Significance was set at a α-level of 0.05.

## Results

### Baseline characteristics

Baseline performance data are presented in Table 2.

**Table 2:** Baseline performance data

	Overall	Males	Females
VO_2peak_ (ml/min/kg)	47 ± 6	51 ± 3	42 ± 2^*^
V_peak_ (km/h)	15.8 ± 1.6	16.7 ± 1.0	14.6 ± 0.9^*^
V_LT_ (km/h)	8.2 ± 0.9	8.5 ± 0.5	8.0 ± 0.7
V_RER_ (km/h)	12.8 ± 1.6	13.8 ± 1.1	11.4 ± 0.4^*^

All values are mean ± SD; *P < 0.05 (gender comparisons only)

### Dietary intake

There were no significant differences (overall and individually) for any of the calculated variables in the reported dietary intake during the 24 h preceding the Fat-peak tests (*P > 0.05*). Mean values for energy, carbohydrate, fat and protein intake were 2507 ± 561 kcal, 345 ± 118 g, 73 ± 34 g and 106 ± 28 g, respectively.

### Fat-peak tests

**Fig. 1:**
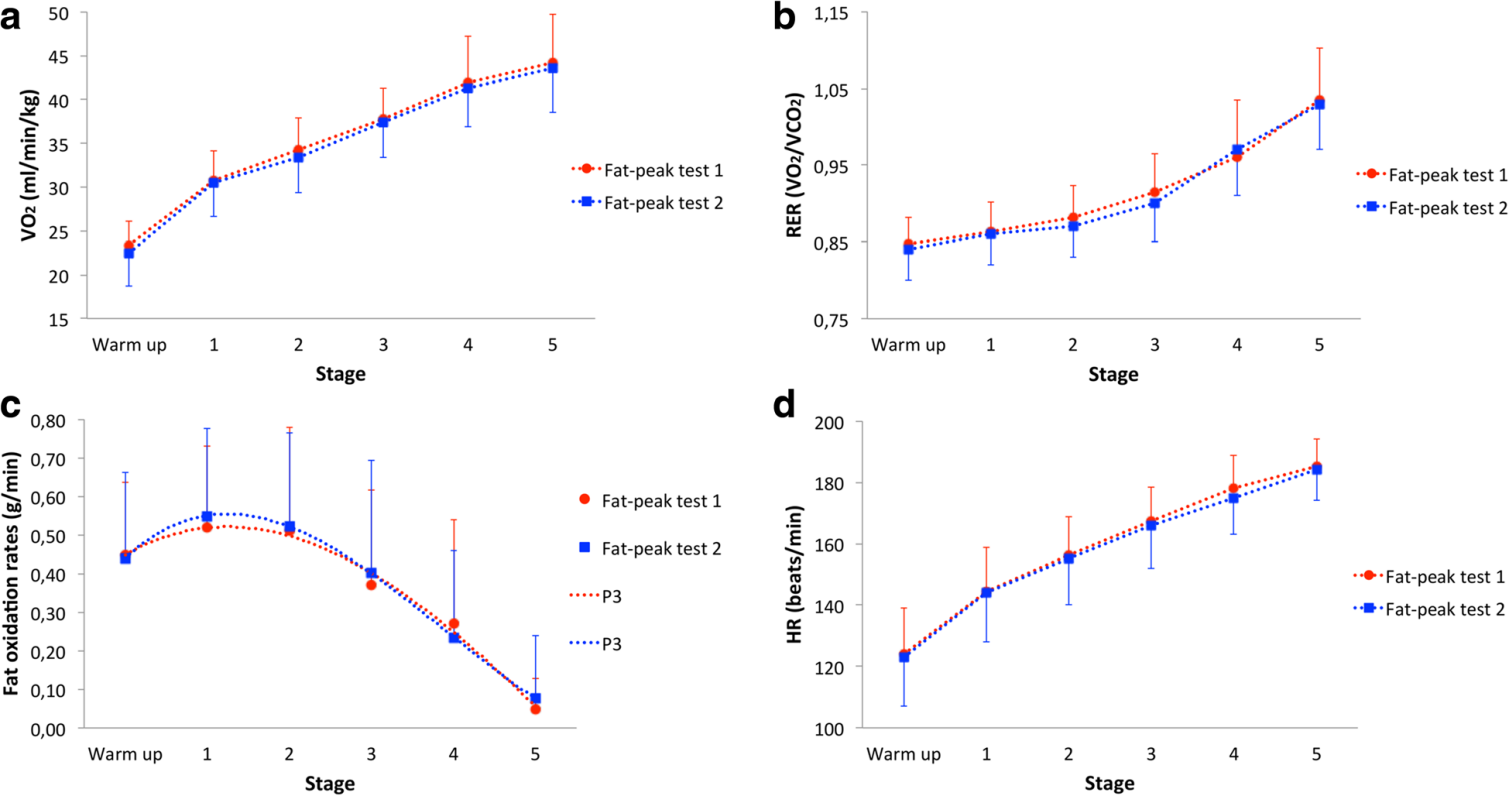
Overview of cardiorespiratory parameters and fat oxidation rates during Fat-peak tests. **a** Average VO_2_ during Fat-peak tests; **b** Average RER during Fat-peak tests; **c** Average fat oxidation rates during Fat-peak tests (P3 interpolated); **d** Average HR during Fat-peak tests. All values are mean ± SD

Individual values for start and end velocities ranged from 6.5 to 10.4 km/h and from 10.9 to 15.6 km/h, respectively. Likewise, stage increments ranged between 0.7 and 1.7 km/h. As shown in Fig. 1 (a-d), there we no significant differences recorded for VO_2_ (*P = 0.20*), RER (*P = 0.58*), fat oxidation rates (*P = 0.79*) and HR (*P = 0.13*) during the two Fat-peak tests. Also with no significant systematic differences between bouts, mean V_PFO_ was 8.2 ± 1.9 and 7.9 ± 1.8 km/h (*P = 0.69*). The range in which individual means of V_PFO_ were detected varied from 5.7 ± 0.2 to 12.5 ± 0.3 km/h, with 11 subjects achieving V_PFO_ (in both tests) during the warm up phase (i.e. below V_LT_). Accordingly, mean PFO was 0.58 ± 0.22 and 0.60 ± 0.22 g/min (*P = 0.85*). The respective range of individual means for PFO went from 0.30 ± 0.08 to 1.03 ± 0.08 g/min. Fat_peak_ averaged at 64 ± 7 and 62 ± 6 % of VO_2peak_ (*P = 0.35*), with individual means ranging from 50 ± 3 to 74 ± 2 % of VO_2peak_. Mean VO_2_ at V_PFO_ was 30 ± 6 and 29 ± 6 ml/min/kg during each of the Fat-peak tests respectively (*P = 0.61*). The corresponding individual means for VO_2_ at V_PFO_ ranged between 21 ± 2 and 40 ± 2 ml/min/kg. Likewise, mean HR at V_PFO_ was 143 ± 11 and 140 ± 13 beats/min (*P = 0.46*), with range of individual means varying between 116 ± 1 and 162 ± 6 beats/min.

### Reliability and day-to-day variability assessment of V_PFO_ and Fat_peak_

**Fig. 2:**
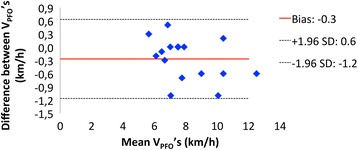
Bland-Altman plot for V_PFO_ during Fat-peak tests

ICC, Pearson’s coefficient and the CV scored 0.98, 0.97 and 5.0 % for V_PFO,_ and 0.90, 0.81 and 7.0 % for Fat_peak_ respectively. As shown in Fig. 2, the bias ± 95 % limits of agreement for V_PFO_ were −0.3 ± 0.9 km/h (−2 ± 8 % of VO_2peak_). Thus, indicating that 95 % of the intra-individual differences should be expected between −1.2 and +0.6 km/h (−10 and +6 % of VO_2peak_).

## Discussion

The aim of the current study was to establish the reproducibility of key parameters that correspond to PFO rates (i.e. V_PFO_ and Fat_peak_) during treadmill ergometry. The main results of this investigation were the high ICC and Pearson’s correlation coefficient computed for V_PFO_ and Fat_peak_, in addition to the correspondingly low CV (i.e. 0.98, 0.97, 5.0 %; and 0.90, 0.81, 7.0 % respectively). Moreover, the performed Bland-Altman analysis has revealed a small bias of −0.3 km/h between Fat-peak tests, with 95 % LoA for the reproducibility of V_PFO_ of 0.9 km/h (i.e. -2 ± 8 % of VO_2peak_).

To our knowledge, the present investigation is the first to report on the reproducibility and day-to-day variability of both V_PFO_ and Fat_peak_ during treadmill ergometry running. Hence, the current results reveal excellent values for the particular relative and absolute reliability indicators. The study group of Gmada [6] seems to be the first to have taken a more comprehensive statistical approach to assess the repeatability of Fat_peak_. In their study, 12 sedentary, but otherwise healthy males performed a graded exercise test (5 stages of 6 min at 20, 30, 40, 50 and 60 % of the maximal aerobic power (MAP)) after a 12-h overnight fast. ICC and CV values for Fat_peak_ across test re-test trials separated by a time interval of 4 days were 0.97 and 5.0 %, respectively. The mean differences ± 95 % LoA for Fat_peak_ was 0.6 ± 7.2 W, indicating that 95 % of the intra-individual differences should be contained between −6.6 and +7.7 W. Based on these values, relative and absolute reliability of Fat_peak_ were deemed as highly reliable by the authors. Unfortunately, no further appraisal has been made to address the physiological plausibility or applicability of the given LoA. Three other investigations have employed similar submaximal graded protocols (i.e. similar stage increment and duration, plus the 12-h overnight food restriction prior to each bout), which were based either on the measured or on the theoretical MAP to establish the reproducibility of Fat_peak_. Yet, conflicting findings have been reported. Pérez-Martin [13] reports a CV of 11.4 % for Fat_peak_, and considered it satisfactory after assessing 10 overweight, but otherwise healthy male participants (no LoA analysis carried out). Similarly, Michallet [14] reports on CV values between 7 and 12 %. Here, the reproducibility of Fat_peak_ was assessed via two different gas exchange techniques in a group of 14 healthy and moderately trained participants (9 males, 5 females). More recently, Croci [16] assessed 15 healthy and moderately trained males, and computed CV values between 16 and 20 % for Fat_peak_ while implementing three different data analysis procedures. The authors additionally report a high intra-individual variability with mean differences ± 95 % LoA for Fat_peak_ (calculated with a P3 function) of −4 ± 32 % of VO_2peak_, indicating that 95 % of the intra-individual differences should be expected between −37 and +28 % of VO_2peak_. Two other investigations using different methodological approaches have addressed the reliability and/or variability of Fat_peak_ estimations. Achten [7] has advocated good reliability after assessing 10 healthy and moderately trained males as they performed an incremental test to exhaustion (test start: 95 W; stage increment and duration: 35 W every 3 min) on three different occasions and after a 12-h overnight fast. The CV for Fat_peak_ (% of VO_2peak_) was 9.6 %. The authors additionally report a root mean square error (typical error) and 95 % confidence interval for Fat_peak_ of 0.23 l/min (0.17 -0.34 l/min). Meyer [15] on the other hand, shows a large intra-individual variability for Fat_peak_ after assessing 21 healthy participants (10 males, 11 females) of varying endurance capacities. Nutrition was moderately controlled, but with no fasting required prior to the exercise bouts. The implemented incremental exercise protocol was nearly identical to the one currently used in our study (further appraisal on the protocol is given below). The mean differences ± 95 % LoA for Fat_peak_ was −13 ± 0.91 l/min (−3.9 ± 28 % of VO_2peak_). Hence, 95 % of intra-individual differences were to be expected between −1.04 and +0.78 l/min (−32 and +23 % of VO_2peak_). In this case, the large variability can be mostly attributed to the fact that only the end of each exercise stage was evaluated and not a continuous curve (i.e. whenever PFO switches from stage 2 to 3, for instance due to a small difference in the recorded rates, it will then result in a large difference in the equivalent % of VO_2peak_).

In the current study, the computed scores agree closely with those reported by Gmada [6], especially the CV, which has come noticeably lower then all of the other values reported in preceding analyses. As to the intra-individual (day-to-day) variability of Fat_peak_, when expressed as % of VO_2peak_, our LoA values have been distinctly lower then those observed by Meyer [15] and Croci [16]. However, whilst these results enable closer comparisons to some of those from other investigations, making reasonable inferences as to the physiological plausibility and practical applicability of these LoA has shown to be a challenging task. As implied by Croci [16], previous studies have deemed an intra-individual variability of ± 10 beats/min for HR at V_PFO_ as acceptable, since this reflects a realistic margin in individuals who use HR for the monitoring of training intensity [7, 15]. Accordingly, in the present investigation this threshold has been sustained in most participants, with only three of them eventually exceeding the given cutoff (though by no more than 3 beats/min). Therefore, based on the strong aggregate of reliability indices and the generally lower intra-individual variability observed for the aforementioned physiological aspects (i.e. Fat_peak_ as % of VO_2peak_ and HR at V_PFO_), we consider the present Fat_peak_ estimations as the most reliable and coherent to date. Furthermore, the employed treadmill running protocol may be used as a reliable tool to identify Fat_peak_ in moderately trained individuals, and according to the reported intra-individual variability values, serve as the basis for future investigational research.

In spite of that, its applicability for athletic training is still questionable. For instance, the high day-to-day variability for PFO (g/min) remains largely unexplained. In the current study, PFO recordings between Fat-peak tests differed by a minimum of 0.01 g/min (1 %) and a maximum of 0.28 g/min (45 %) among the participants, which is consistent with inter- and intra-individual patterns described in previous investigations [1, 15, 16]. On the grounds of this known variability for PFO, recent studies [25, 26] have questioned the practical applicability of prescribing exercise training based on Fat_peak_, since it remains debatable whether prolonged exercise at Fat_peak_ can indeed be maintained with PFO rates. Therefore, it may be ultimately necessary for prospective studies (e.g. those looking at the sustainability of PFO during prolonged exercise bouts at Fat_peak_) to consider the LoA (or simply the individual test re-test difference) for Fat_peak_, V_PFO_ and PFO. Then, based on that, delineate the ± intensities in which exercise bouts should be performed and eventually evaluate how this impacts the sustainability of PFO (i.e. also in accordance to the identified intra-individual variability of each person). Other questions in need of further research include: 1) What are the physiological determinants and additional intrinsic/extrinsic factors influencing the variability of fat oxidation rates during running, as well as in other types exercise? 2) How applicable, versatile and reliable is the current protocol across different cohorts of people (e.g. patients, untrained persons or professional athletes)?

To date, there have been a few investigations assessing the reproducibility of Fat_peak_ [6, 7, 13–16]. Though the majority of those have failed to make thorough statistical analyses by not providing indicators of both relative and absolute reliability for Fat_peak_ estimations (i.e. the degree to which individuals/variables maintain their position in a sample with repeated measurements; or the degree to which repeated measurements vary for individuals/variables), in addition to practical information on the respective intra-individual (day-to-day) variability by establishing the LoA (i.e. the individual subject differences in a test re-test plotted against the respective individual means) [18, 27–29]. Hereto, previous studies suggest that an ICC greater than 0.90 is reflective of high relative reliability, while values between 0.80 and 0.90 should be rated as moderate, with figures under 0.80 being graded as not sufficient for physiological testing [6, 30]. Additionally, a Pearson’s coefficient greater than 0.80 is advocated as high [18], whereas a CV under 10 % can be considered as an indicator for a reliable test, being a commonly used and accepted threshold for biological variables [6, 31, 32].

In the current study we have implemented rigid pre-testing conditions with standardized nutrition and exercise restraint for the 24 h prior to each submaximal bout. Yet, other methodological factors such as the elected exercise protocol, data analysis approach as well as the embedded equipment error may affect the determination of fat oxidation rates and subsequently V_PFO_ [16]. The currently employed exercise protocol intends to cover the realistic range for V_PFO_ determination and takes into account important physiological aspects in its design to ensure gas exchange maintains steady state for as long as possible [15]. The start velocity (V_LT_) corresponds to the first increase in blood lactate and can be considered as the upper border for the conduction of regenerative training. The end velocity (V_RER_) represents a metabolic state where energy supply is expected to yield solely from carbohydrate metabolism. Ultimately, three stages in between these metabolic markers should account for an accurate determination of V_PFO_ [15, 21, 33, 34]. Additionally, we have chosen to create P3 curves, as it is a valid and widely used method that models the overall kinetics of fat oxidation for a more coherent representation of V_PFO_ and PFO [12].

Here we would like to comment on the 11 participants that had their V_PFO_ and Fat_peak_ computed during the warm up phase. One reason for this could of course be the rather moderate aerobic endurance capacity of participants, since in less trained individuals Fat_peak_ occurs at lower exercise intensities than in trained individuals [34]. However, when looking at the individual raw fat oxidation rates, only 5 subjects have had indeed higher fat oxidation values during the warm up phase. The remaining 6, had their highest raw values recorded at the end of the first stage and were somewhat “drifted backwards” due to the applied P3 interpolation and how the curve-fit reacted upon the variables. Such a drift can also occur in the opposite way as depicted in Fig. 1c, which in this case, was caused when curve-fitting the overall means for fat oxidation rates instead of individual values. This prompted the curve into a small elongation (likely driven by the subjects that had PFO rates at the latter stages of the tests). Hence, the depiction of PFO rates that are slightly lower than the mean of individually interpolated values, and which also occur during the test phase and not the warm up. Still, the use of a mathematical model such as the P3, is a more consistent approach than just accounting for the raw measured values when analyzing data that does not align in a perfect curve [12]. However, alternative ways of curve-fitting might be evaluated in the future.

At last, it must be noted that the total variation observed in our test re-test is a sum of both biological and equipment variation (error) [15, 16]. Though analyzing the relative contribution of each of these parameters was beyond the scope of this study, the used gas exchange analyzer has been considered reliable [35]. Ideal ICC values (1.00) were computed for ventilation (V_E_) VO_2_ and VCO_2_. Respectively, the average intra-device technical error of measurement (%TEM) was 0.2, 1.4 and 1.1 %.

## Conclusion

The present study for the first time aimed at investigating the reliability and day-to-day variability of peak fat oxidation in treadmill running in moderately trained male and female recreational athletes, using appropriate statistical methods. In summary, the reproducibility of V_PFO_ and Fat_peak_ during treadmill ergometry was found to be excellent with ICC, Pearson’s correlation coefficient and CV scoring at 0.98, 0.97, 5.0 %; and 0.90, 0.81, 7.0 % respectively. Fat_peak_ determined in a treadmill test might therefore serve as training prescription, although fat oxidation rates at prolonged exercise bouts at this intensity still need to be investigated.

## Abbreviations

VO_2peak_: Peak oxygen uptake
PFO: Peak fat oxidation
Fat_peak_: Intensity eliciting peak fat oxidation rates
V_PFO_: Velocity at which peak fat oxidation occurs
BMI: Body mass index
%BF: Percentage body fat
V_LT_: Velocity at aerobic threshold
RER: Respiratory exchange ratio
V_RER_: Velocity at respiratory exchange ratio of 1.00
V_peak_: Peak running velocity
VO_2_: Oxygen uptake
HR: Heart rate
P3: Third polynomial
ICC: Intraclass correlation coefficient
CV: Coefficient of variation
Bias: Mean differences
LoA: Limits of agreement
MAP: Maximal aerobic power
%TEM: Technical error of measurement
